# High-Salt Diet Exacerbates Murine Imiquimod-Induced Psoriasis-like Dermatitis via Activation of Serum- and Glucocorticoid-Inducible Kinase 1

**DOI:** 10.3390/ijms27188151

**Published:** 2026-09-13

**Authors:** Mai Yoshida, Teppei Hagino, Manami Yoneyama, Hidehisa Saeki, Naoko Kanda

**Affiliations:** 1 Department of Dermatology, Nippon Medical School, Bunkyo City 113-8602, Tokyo, Japanh-saeki@nms.ac.jp (H.S.); 2 Department of Dermatology, Nippon Medical School Chiba Hokusoh Hospital, Inzai 270-1694, Chiba, Japan; teppei-hagino@nms.ac.jp (T.H.);

**Keywords:** psoriasis, high-salt diet, imiquimod, IL-17, IL-23, serum- and glucocorticoid-inducible kinase 1

## Abstract

Psoriasis is a chronic skin disease manifesting scaly, indurated erythema and an enhanced interleukin (IL)-23/IL-17 immune axis. A high-salt diet (HSD) is known to promote several autoimmune or autoinflammatory diseases. We examined whether an HSD might exacerbate topical imiquimod (IMQ)-induced psoriasis-like dermatitis in mice. Balb/c female mice were fed a normal diet (ND, 0.2% NaCl) or an HSD (4.2% NaCl) for 3 weeks, and 25 mg IMQ was applied to shaved back skin for 5 consecutive days. Compared to ND, the HSD exacerbated IMQ-induced psoriasiform dermatitis with increased scale, skin thickness, and total scores, and histologically enhanced epidermal thickening, hyperkeratosis, and dermal inflammatory infiltrates, including Ly6G+ neutrophils and CD3+ T cells. HSD increased mRNA levels of *Il17a*, *Il22*, *Tnfa*, *Il1b*, *Il23a*, *Il23r*, *Cxcl1*, *Ccl20*, *Krt16*, and *Ccnd1* in IMQ-induced dermatitis. HSD increased the immunostaining areas of whole and Ser 422-phosphorylated forms of serum- and glucocorticoid-inducible kinase 1 (SGK1) in epidermal keratinocytes and dermal-infiltrating cells in IMQ-induced dermatitis. Intraperitoneal SGK1 inhibitor EMD638683 reversed the above effects of HSD in exacerbating IMQ-induced dermatitis. Our present results indicate that HSD may exacerbate murine IMQ-induced psoriasis-like dermatitis at least partially through the activation of SGK1.

## 1. Introduction

Psoriasis is a recurrent inflammatory skin disease manifesting erythema, scales, and skin thickening. Psoriasis is characterized by an enhanced interleukin (IL)-23/IL-17 immune axis, exaggerated proliferation of epidermal keratinocytes, and prominent inflammation [1]. In the onset of psoriasis, dendritic cells (DCs) triggered by certain stimuli secrete tumor necrosis factor-α (TNF-α), which stimulates themselves and promotes their IL-23 secretion. IL-23 induces type 17 helper T (Th17) cells to produce IL-17A and IL-22, which act on keratinocytes to induce their proliferation and secretion of inflammatory cytokines or chemokines that attract neutrophils, monocytes, and lymphocytes. Innate immune cells, such as type 3 innate lymphoid cells, γδT cells, or mucosal-associated invariant T cells, also secrete IL-17A, which promotes the development of psoriasis [2,3]. Regulatory T cells (Tregs) inhibit the proliferation of pathogenic Th17 cells. The number and/or function of Tregs are reduced in psoriasis patients compared to healthy subjects, which then fails to sufficiently suppress the activity of pathogenic Th17 cells [4,5,6]. A variety of environmental factors can trigger or worsen psoriasis [2]. Diet is one such environmental factor. It is reported that a Western diet characterized by high fat, high sugar, high salt, and low fiber may promote the development or exacerbation of psoriasis [7], while an inulin dietary fiber-supplemented diet improved murine psoriasis-like dermatitis [8]. We found that dietary intake of salt was increased in patients with palmoplantar pustulosis, a disease related to psoriasis, compared to healthy individuals [9]. Research on the UK Biobank cohort of 400,000 participants showed that the risk of psoriasis increases with increasing frequency of adding salt to foods [10], indicating the stimulatory effect of high-salt intake on psoriasis.

Extracellular sodium is transported via epithelial sodium channels (ENaCs) into antigen-presenting cells (APCs) such as DCs or monocytes/macrophages, and the sodium entry activates cytosolic serum- and glucocorticoid-inducible kinase 1 (SGK1), which further induces robust sodium entry via ENaCs [11]. The sodium entry results in the generation of reactive oxygen species (ROS) and isolevuglandin (isoLG), an oxidative product of arachidonic acid metabolism, and the activation of nucleotide-binding oligomerization domain-like receptor family pyrin domain containing 3 (NLRP3) inflammasome, and these mediators promote the production of IL-1β or IL-23 in APCs [11,12,13]. It is reported that a high-salt diet (HSD) promotes the development of pathogenic Th17 cells [14,15,16,17] while down-regulating Treg activity in mice and humans [18,19]. High extracellular sodium acts on naïve T cells and increases *IL23r* expression, while decreasing *Foxp3* expression via SGK1 [20,21]. It is reported that HSD exacerbates murine experimental allergic encephalomyelitis [16] and autoimmune arthritis [17].

In this study, we investigated whether HSD might exacerbate psoriasis-like dermatitis induced by topical application of imiquimod (IMQ), a Toll-like receptor 7 agonist, on the skin of mice, with particular focus on the involvement of SGK1. The results showed that HSD exacerbated IMQ-induced psoriasis-like dermatitis clinically, with increased scaling and skin thickening, and histologically, with the enhancement of hyperkeratosis, acanthosis, and dermal inflammatory infiltration, including that of T cells and neutrophils. Furthermore, the results showed increased mRNA levels of IL-17-related inflammatory or proliferative markers, *Il17a*, *Il22*, *Il23a*, *Il23r*, *Il1b*, *Tnfa*, *Cxcl1*, *Ccl20*, *Krt16*, and *Ccnd1*, together with increased immunostaining areas of whole and active forms of SGK1 in lesional skin, and that the intraperitoneal administration of SGK1 inhibitor EMD638683 reversed such effects of HSD. The results indicate that HSD might exacerbate murine IMQ-induced psoriasis-like dermatitis at least partially through SGK1.

## 2. Results

### 2.1. HSD Exacerbated Murine IMQ-Induced Psoriasis-like Dermatitis

#### 2.1.1. HSD Increased Skin Thickness, Scale, and Total Scores of IMQ-Induced Dermatitis

**Figure 1:**
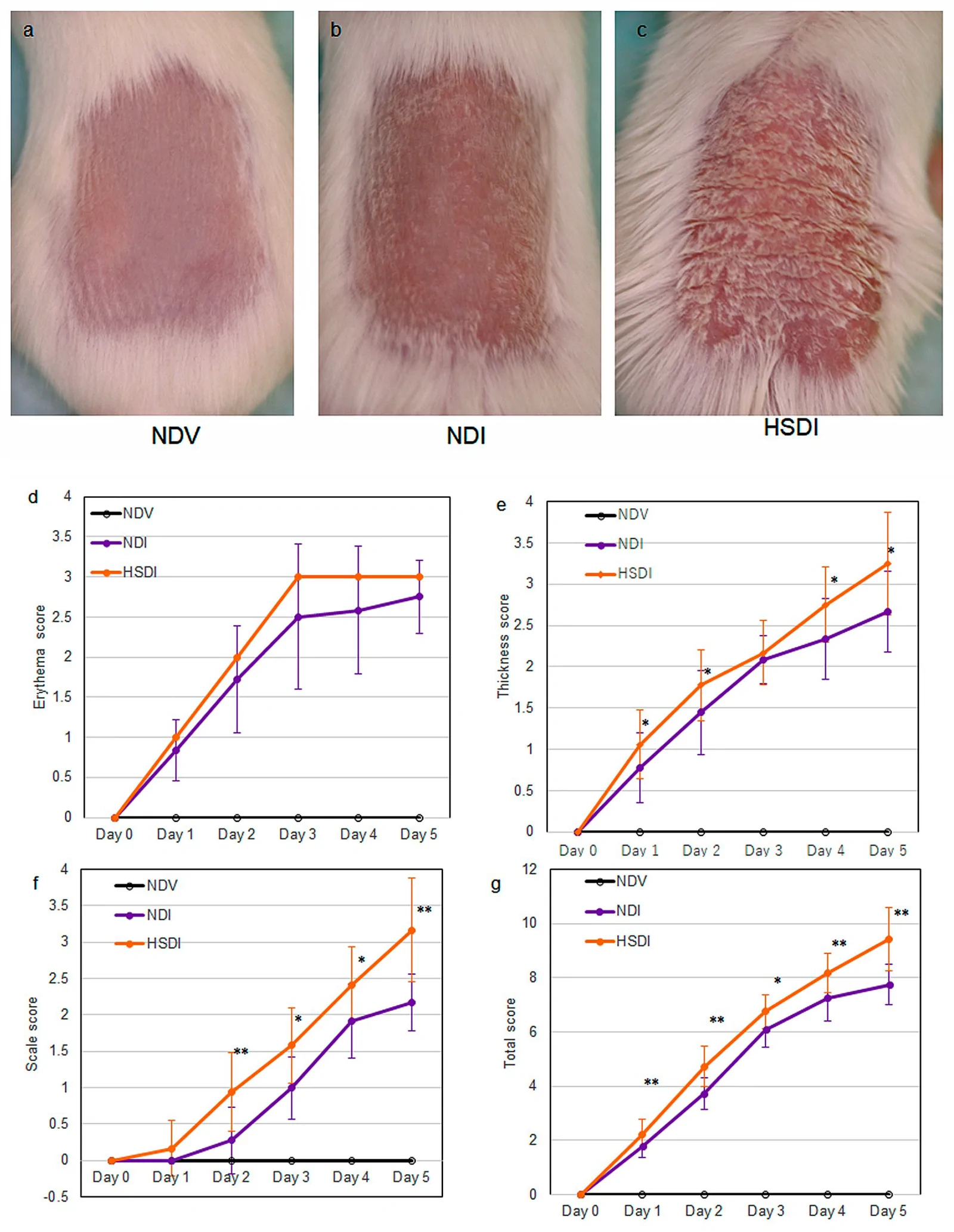
High-salt diet (HSD) exacerbates imiquimod (IMQ)-induced psoriasis-like dermatitis. Photos presenting shaved back skin of normal diet (ND)-fed, Vaseline-treated mice (NDV) (**a**); ND-fed, IMQ-treated mice (NDI) (**b**); and HSD-fed, IMQ-treated mice (HSDI) (**c**). The erythema (**d**), thickness (**e**), scale (**f**), and total scores (**g**) are presented as mean ± standard deviation (*n* = 12/group). * *p* < 0.05, ** *p* < 0.01 versus NDI, by one-way ANOVA, followed by Tukey post hoc test.

Topical application of IMQ 25 mg on shaved back skin induced a psoriasis-like rash with skin thickening, erythema, and scales (Figure 1b,d–g, purple lines), while these features were not found in Vaseline-treated mice (Figure 1a,d–g, black lines). Compared to an ND, feeding mice an HSD significantly increased thickness, scale, and total scores in IMQ-induced dermatitis (Figure 1c,e–g, orange lines), while there were no significant differences in erythema scores (Figure 1d).

#### 2.1.2. HSD Enhanced Acanthosis, Hyperkeratosis, and Infiltration of Inflammatory Cells in IMQ-Induced Dermatitis

**Figure 2:**
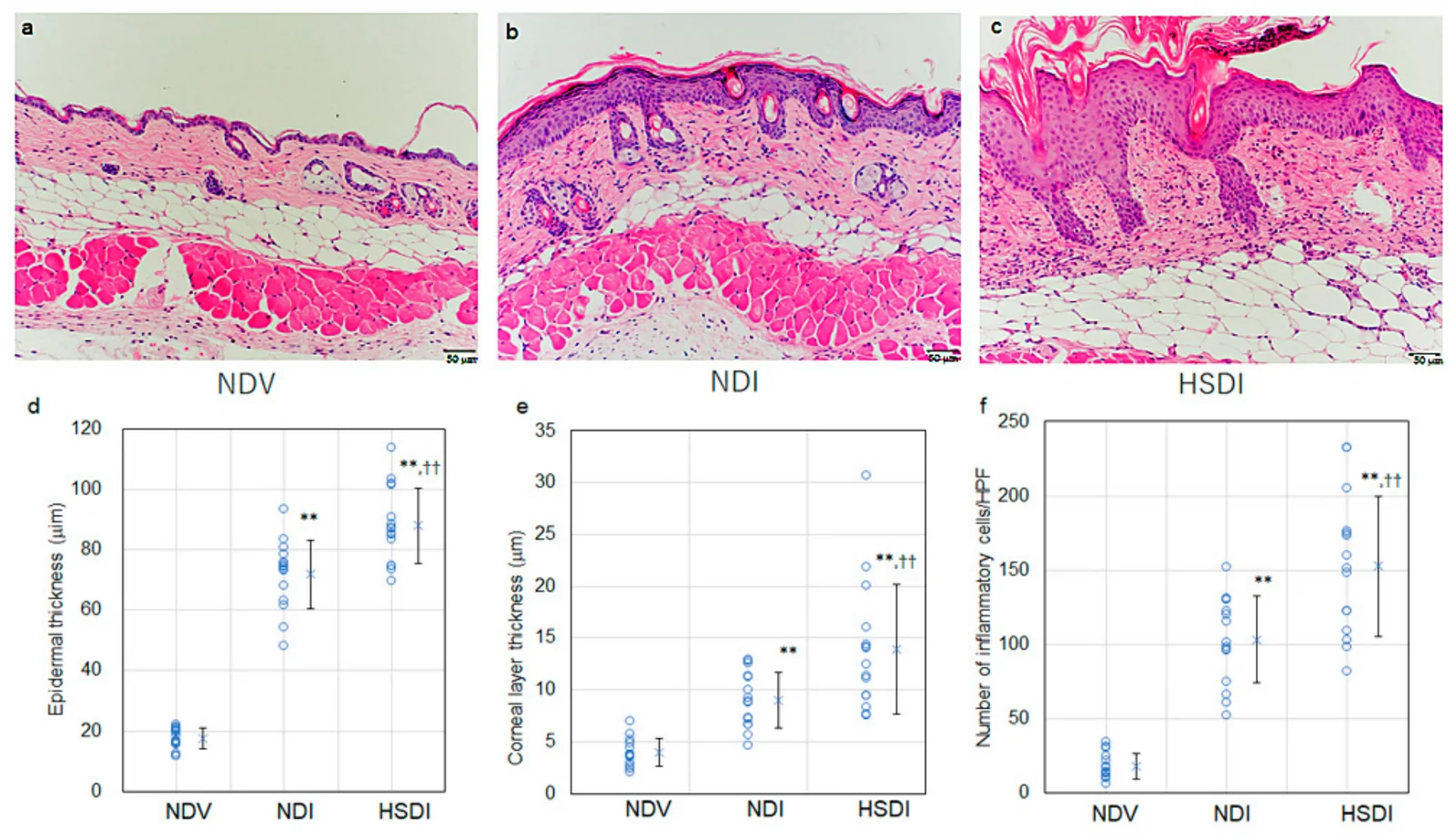
High-salt diet (HSD) promotes acanthosis, hyperkeratosis, and inflammatory infiltration in imiquimod (IMQ)-induced dermatitis. Hematoxylin–eosin-stained skin sections from normal diet (ND)-fed, Vaseline-treated mice (NDV) (**a**); ND-fed, IMQ-treated mice (NDI) (**b**); and HFD-fed, IMQ-treated mice (HSDI) (**c**). The bar in each panel represents 50 μm. The thickness of the epidermis (**d**) and of corneal layers (**e**), and the number of infiltrating cells (**f**) measured or counted under × 400 high-power field (HPF) in five random grids per section are presented as individual values with mean ± standard deviation (*n* = 15/group). ** *p* < 0.01, versus NDV; †† *p* < 0.01 versus NDI, by one-way ANOVA, followed by Tukey post hoc test.

The IMQ-treated skin of ND-fed mice manifested psoriasis-like histology showing epidermal thickening with elongation of rete ridges, hyperkeratosis, parakeratosis, and abundant infiltration of inflammatory cells in the dermis (Figure 2b,d–f, middle bars). HSD further increased the thickness of the epidermis and corneal layers, and the number of infiltrating inflammatory cells in IMQ-treated skin (Figure 2c,d–f, right bars).

**Figure 3:**
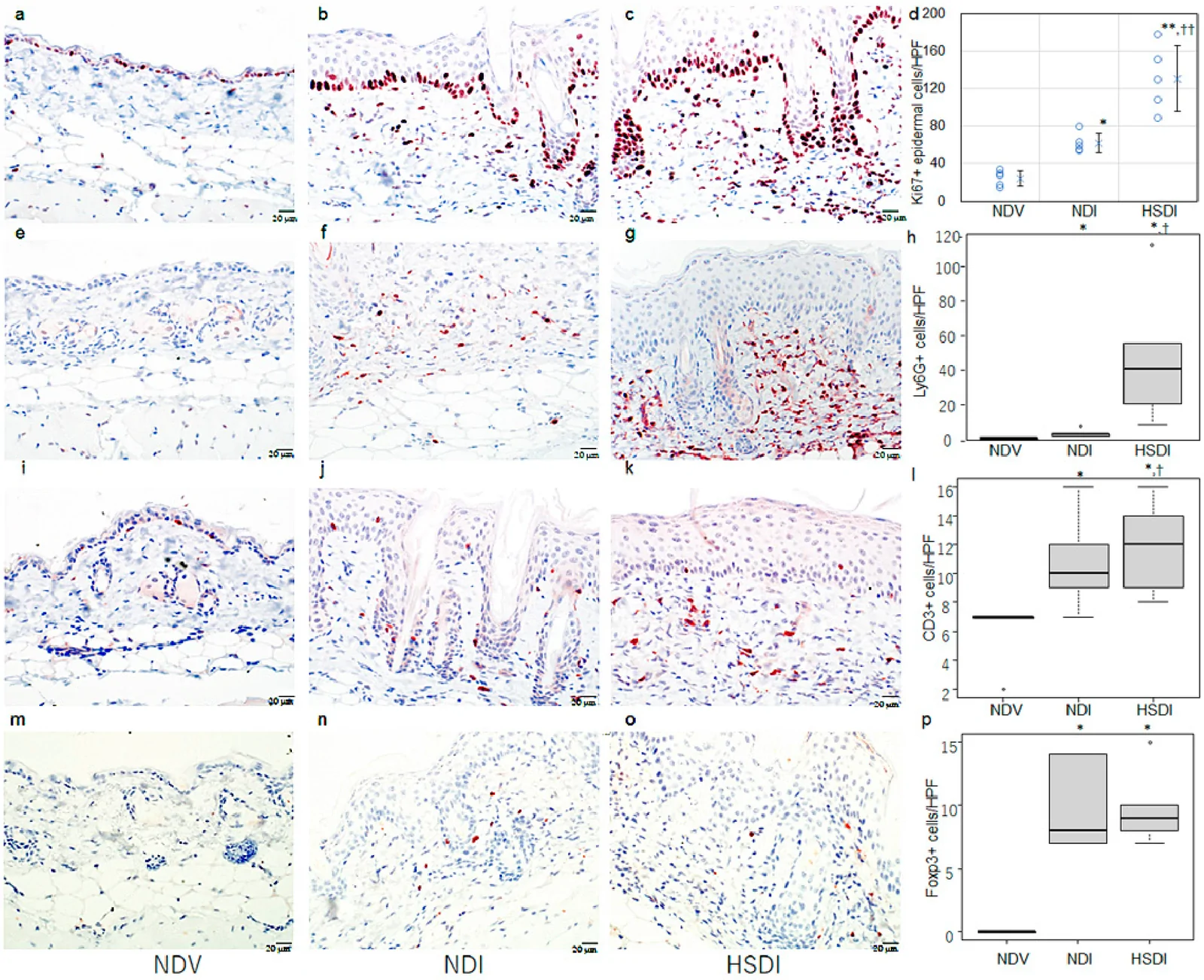
High-salt diet (HSD) increases the numbers of epidermal Ki67+ cells, Ly6G+ neutrophils, and CD3+ T cells in imiquimod (IMQ)-induced dermatitis. Skin sections from normal diet (ND)-fed, Vaseline-treated mice (NDV) (**a**,**e**,**i**,**m**); ND-fed, IMQ-treated mice (NDI) (**b**,**f**,**j**,**n**); and HSD-fed, IMQ-treated mice (HSDI) (**c**,**g**,**k**,**o**) were immunohistochemically stained for Ki67 (**a**–**c**), Ly6G (**e**–**g**), CD3 (**i**–**k**), and Foxp3 (**m**–**o**). The bar in each panel represents 20 μm. The numbers of cells positive for Ki67 (**d**) are presented as individual vales with mean ± standard deviation, while those for Ly6G (**h**), CD3 (**l**), or Foxp3 (**p**) are presented as median [interquartile range]. The number of each marker-positive cells was counted under × 400 high-power field (HPF) in five random grids per section (*n* = 5/group). * *p* < 0.05, ** *p* < 0.01, versus NDV; † *p* < 0.05, †† *p* < 0.01 versus NDI, by one-way ANOVA, followed by Tukey post hoc test (**d**) or by Kruskal–Wallis test followed by Steel Dwass post hoc test (**h**,**l**,**p**).

Topical IMQ increased the number of epidermal Ki67+ proliferating cells, which was further increased by HSD (Figure 3a–d). Topical IMQ increased the number of infiltrating Ly6G+ neutrophils, which was further increased by HSD (Figure 3e–h). Topical IMQ increased the number of CD3+ T cells, which was moderately increased by HSD (Figure 3i–l). Topical IMQ increased the number of Foxp3+ cells in the skin, and the number was not significantly altered by HSD (Figure 3m–p). Those findings suggest that HSD may increase the number of proliferating keratinocytes and infiltrating neutrophils and T cells in IMQ-induced psoriasis-like dermatitis.

#### 2.1.3. HSD Increased mRNA Levels of Proinflammatory Cytokines/Chemokines and Proliferation-Related Molecules

**Figure 4:**
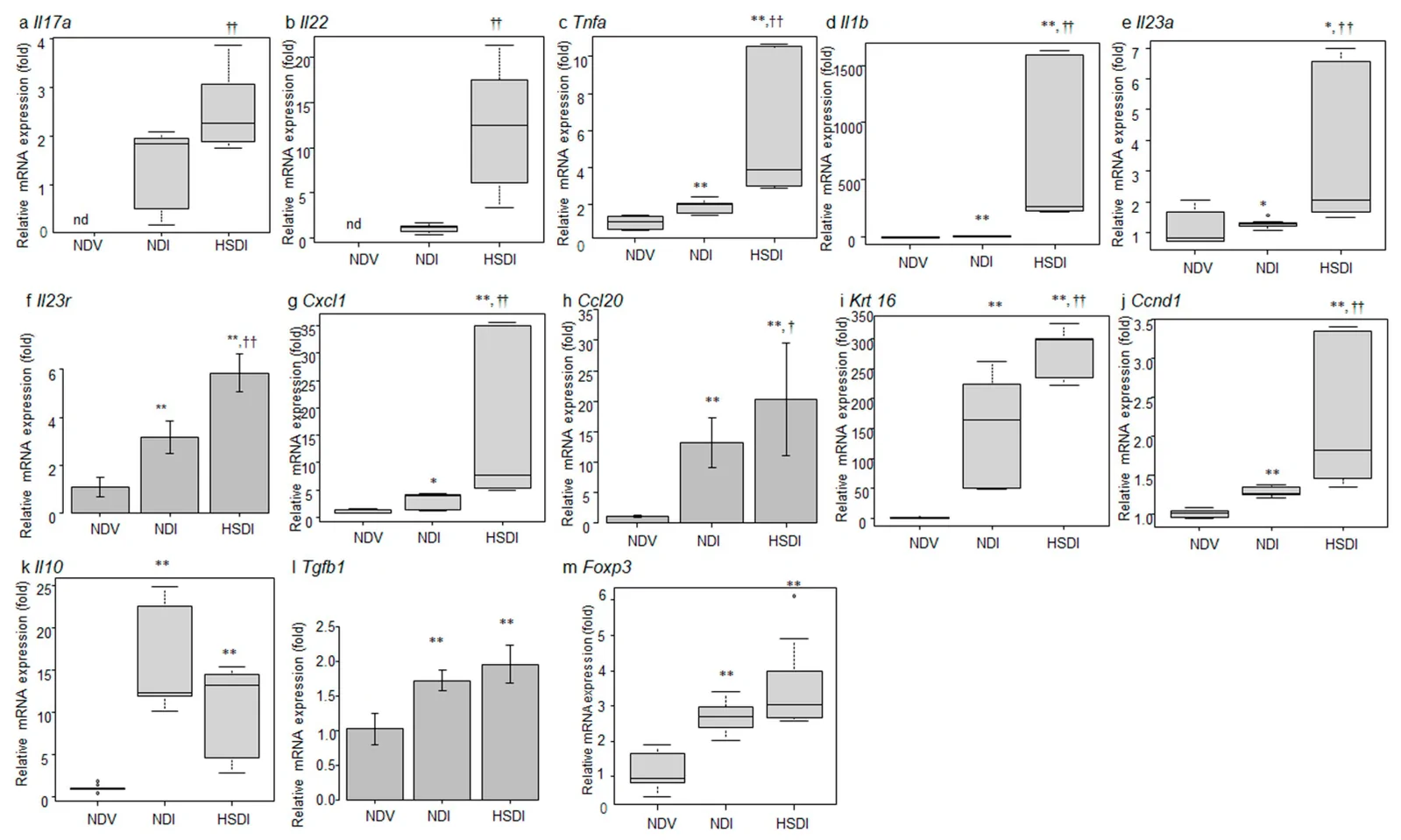
mRNA levels of *Il17a* (**a**), *Il22* (**b**), *Tnfa* (**c**), *Il1b* (**d**), *Il23a* (**e**), *Il23r* (**f**), *Cxcl1* (**g**), *Ccl20* (**h**), *Krt16* (**i**), *Ccnd1* (**j**), *IL10* (**k**), *Tgfb1* (**l**), and *Foxp3* (**m**) in the skin from normal diet (ND)-fed, Vaseline-treated mice (NDV); ND-fed, imiquimod (IMQ)-treated mice (NDI); and high-salt diet-fed, IMQ-treated mice (HSDI); all evaluated by qPCR. mRNA levels of individual molecules normalized to those of *Gapdh* are shown as fold induction relative to that of NDV, presented as mean ± standard deviation or median [interquartile range] (*n* = 9/group). *Il17a* and *Il22* mRNAs normalized to that of *Gapdh* were not detected in NDV, and their levels are presented as fold induction relative to NDI. * *p* < 0.05, ** *p* < 0.01, versus NDV; † *p* < 0.05, †† *p* < 0.01 versus NDI, by Kruskal–Wallis test followed by Steel Dwass post hoc test (**a**–**e**,**g**,**i**–**k**,**m**), or by one-way ANOVA followed by Tukey post hoc test (**f**,**h**,**l**). nd, not detected.

We analyzed the effects of HSD on steady-state mRNA levels of Th17-related pro-inflammatory cytokines/chemokines or proliferation-related molecules in IMQ-induced dermatitis by qPCR. Compared to the Vaseline control (Figure 4a–l, left bars), topical IMQ increased mRNA levels of Th17-related proinflammatory cytokines *Il17a*, *Il22*, *Tnfa*, and *Il1b*; *Il23a* and its receptor *Il23r*; and chemokines *Cxcl1* and *Ccl20* in the skin of ND-fed mice (Figure 4a–h, middle bars). The HSD further increased mRNA levels of these molecules induced by IMQ (Figure 4a–h, right bars). Compared to the Vaseline control, topical IMQ also increased mRNA levels of proliferation-associated cytokeratin *Krt16* and cell cycle progressor *Ccnd1* in the skin of ND-fed mice, and these mRNA levels were further increased by HSD (Figure 4i,j). Compared to the Vaseline control, topical IMQ increased mRNA levels of anti-inflammatory cytokines *Il10* and *Tgfb1*, and Treg marker *Foxp3*, whose levels were not significantly altered by HSD (Figure 4k–m). These results indicate that HSD increased mRNA levels of Th17-related pro-inflammatory or growth-stimulatory molecules in IMQ-induced dermatitis. These effects might be related to the HSD-induced exacerbation of acanthosis, hyperkeratosis, dermal inflammatory infiltration, and severity of dermatitis.

### 2.2. HSD Increased Immunostaining Areas of Whole and Activated Forms of SGK1 in IMQ-Induced Dermatitis

**Figure 5:**
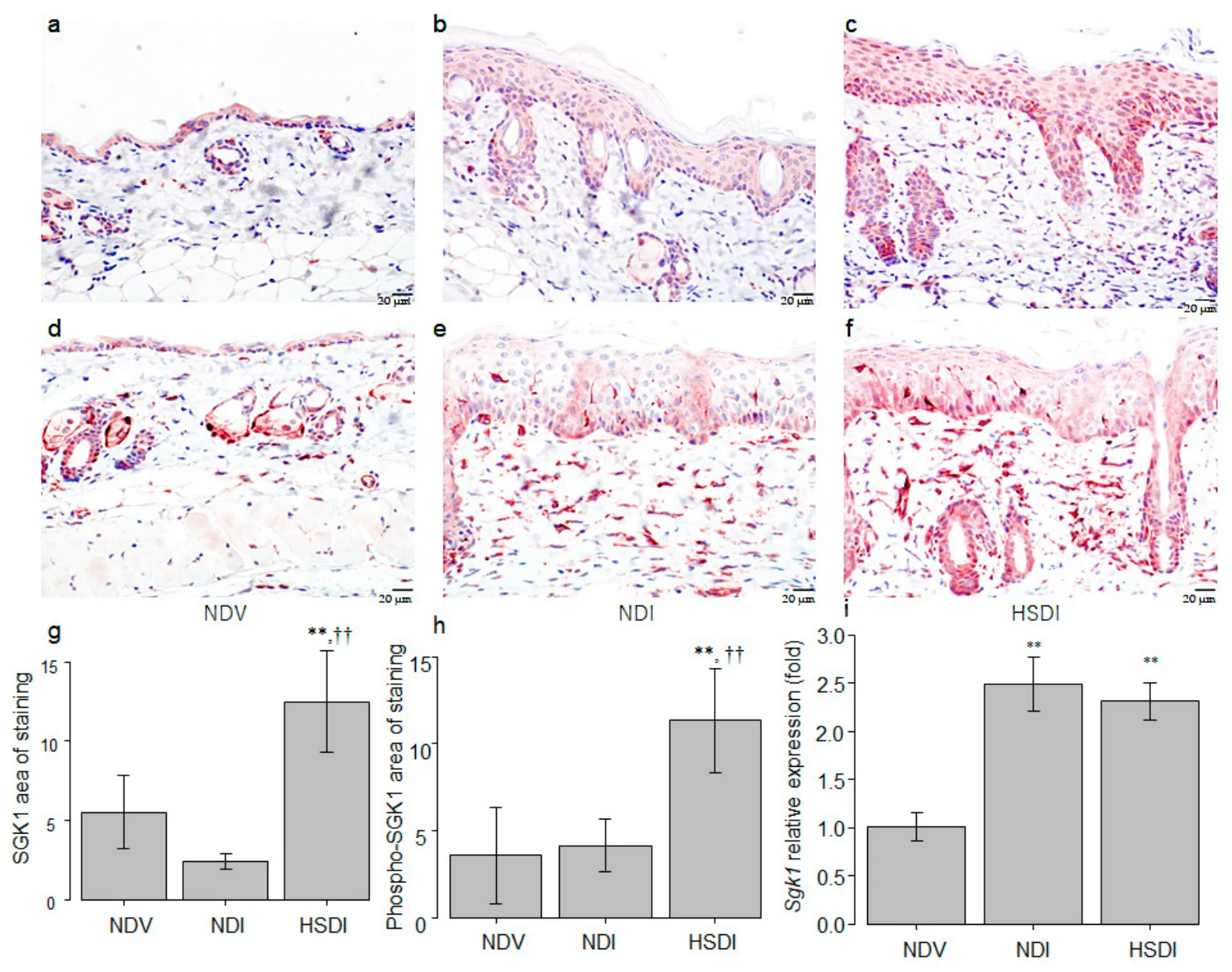
High-salt diet (HSD) increases the immunostaining areas of whole and Ser 422-phosphorylated forms of SGK1 in imiquimod (IMQ)-induced dermatitis. Skin sections from normal diet (ND)-fed, Vaseline-treated mice (NDV) (**a**,**d**); ND-fed, IMQ-treated mice (NDI) (**b**,**e**); and HSD-fed, IMQ-treated mice (HSDI) (**c**,**f**) were immunohistochemically stained for SGK1 (**a**–**c**) or phospho-SGK1 (**d**–**f**). The bar in each panel represents 20 μm. The area of immunostaining for SGK1 (**g**) or phospho-SGK1 (**h**) is presented as mean ± standard deviation (*n* = 5/group). ** *p* < 0.01 versus NDV; †† *p* < 0.01 versus NDI, by one-way ANOVA, followed by Tukey post hoc test. *Sgk1* mRNA levels in the skin were evaluated by qPCR. mRNA levels of *Sgk1* normalized to those of *Gapdh* are shown as fold induction relative to that of NDV, presented as mean ± standard deviation (**i**) (*n* = 9/group).

It has been reported that high sodium promotes the expression and/or activity of SGK1, and that activated SGK1 may contribute to the exacerbation of Th17-related inflammation [20,21,22]. We thus examined the expression of whole and activated forms of SGK1 in IMQ-induced dermatitis of mice fed with an ND or HSD, by immunohistochemical analysis. Topical IMQ did not significantly alter the immunostaining areas of whole (Figure 5a–c,g) and Ser 422-phosphorylated (activated) forms of SGK1 (Figure 5d–f,h) in the skin compared to the Vaseline control, while the HSD group demonstrated increased immunostaining areas of both forms of SGK1, mainly in epidermal keratinocytes and dermal-infiltrating cells of IMQ-treated skin. Topical IMQ increased mRNA levels of *Sgk1*, and an HSD did not further increase the mRNA level (Figure 5i). These results indicate that an HSD may increase immunostaining areas of whole and activated SGK1 without altering its mRNA levels in IMQ-induced dermatitis.

### 2.3. SGK1 Inhibitor EMD638683 Reversed HSD-Induced Exacerbation of IMQ-Induced Dermatitis

#### 2.3.1. The Inhibitory Effects of Intraperitoneal EMD638683 on Clinical and Histological Findings Exacerbated by HSD

**Figure 6:**
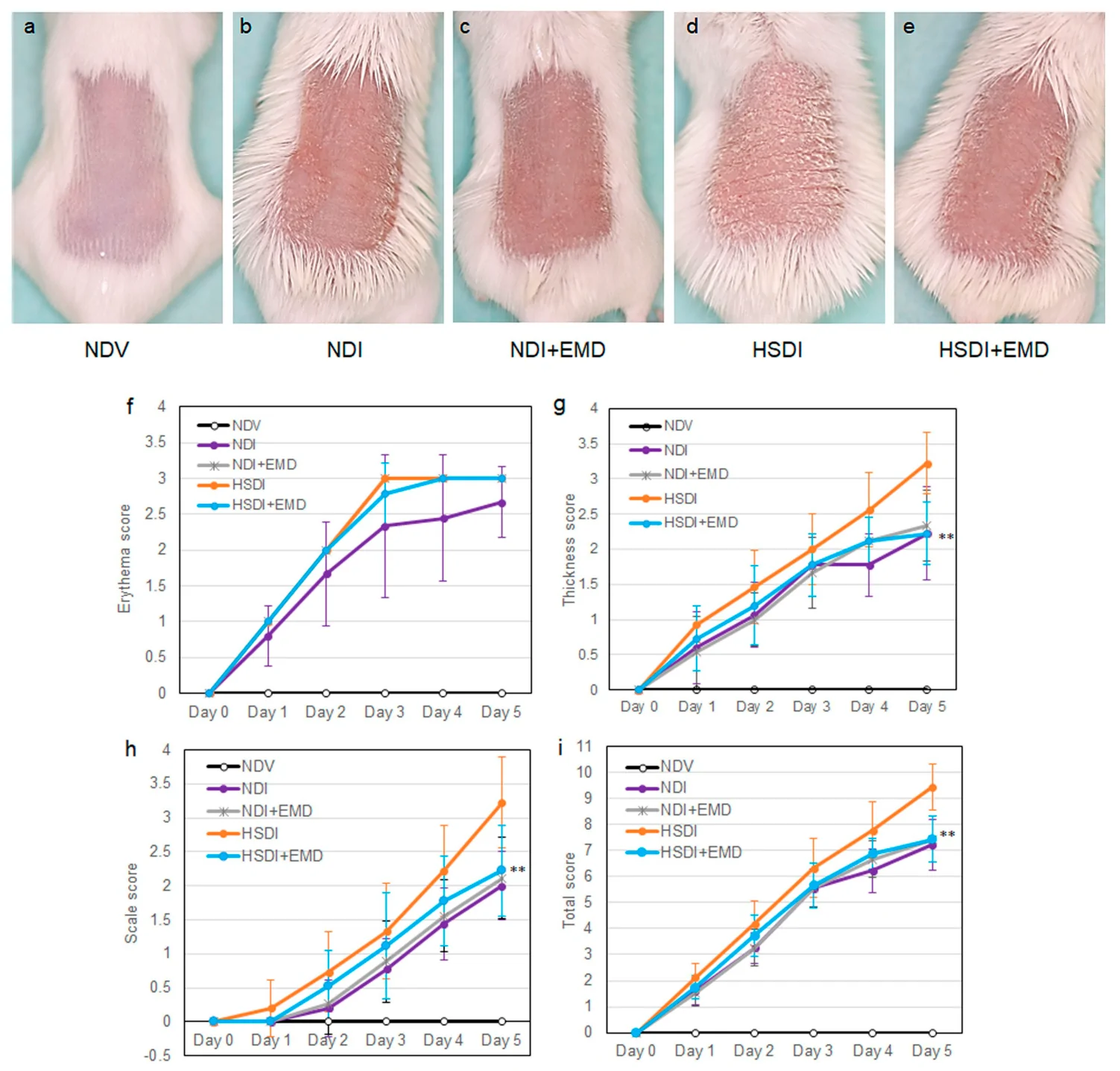
Photos of shaved back skin of normal diet (ND)-fed, Vaseline-treated mice intraperitoneally injected with 1% dimethyl sulfoxide in phosphate-buffered saline (DMSO-PBS) (NDV) (**a**); ND-fed, imiquimod (IMQ)-treated, DMSO-PBS-injected mice (NDI) (**b**); ND-fed, IMQ-treated mice injected with EMD638683 (EMD) in DMSO-PBS (NDI + EMD) (**c**); high-salt diet (HSD)-fed, IMQ-treated, DMSO-PBS-injected mice (HSDI) (**d**); and HSD-fed, IMQ-treated mice injected with EMD in DMSO-PBS (HSDI + EMD) (**e**). The erythema (**f**), thickness (**g**), scale (**h**), and total scores (**i**) are presented as mean ± standard deviation (*n* = 9/group). ** *p* < 0.01 in comparison of HSDI + EMD (light blue) versus HSDI (orange), by one-way ANOVA, followed by Tukey post hoc test.

HSD-induced increases in immunostaining area for whole and active forms of SGK1 indicate that SGK1 may mediate the HSD-induced exacerbation of IMQ-induced dermatitis. We thus examined whether the SGK1 inhibitor EMD638683 might attenuate the HSD-induced exacerbation of IMQ-induced dermatitis. Mice fed with an ND or HSD were intraperitoneally administered EMD638683 or a control vehicle, then IMQ was topically applied to the skin, and the clinical and histological findings of dermatitis were compared. Intraperitoneal EMD638683 significantly decreased skin thickness, scale, and total scores of dermatitis in HSD-fed, IMQ-treated mice (HSDI) mostly to the levels of ND-fed and IMQ-treated mice (NDI) on Day 5 (Figure 6d,e,g–i, orange versus light blue lines), while there were no statistically significant differences in these scores throughout Days 1 to 4. On the other hand, EMD638683 did not appear to reduce these scores in NDI (Figure 6b,c,g–i, purple versus gray lines). These results indicate that SGK1 may mediate HSD-induced exacerbation of IMQ-induced dermatitis but may not be involved in IMQ-induced dermatitis itself.

**Figure 7:**
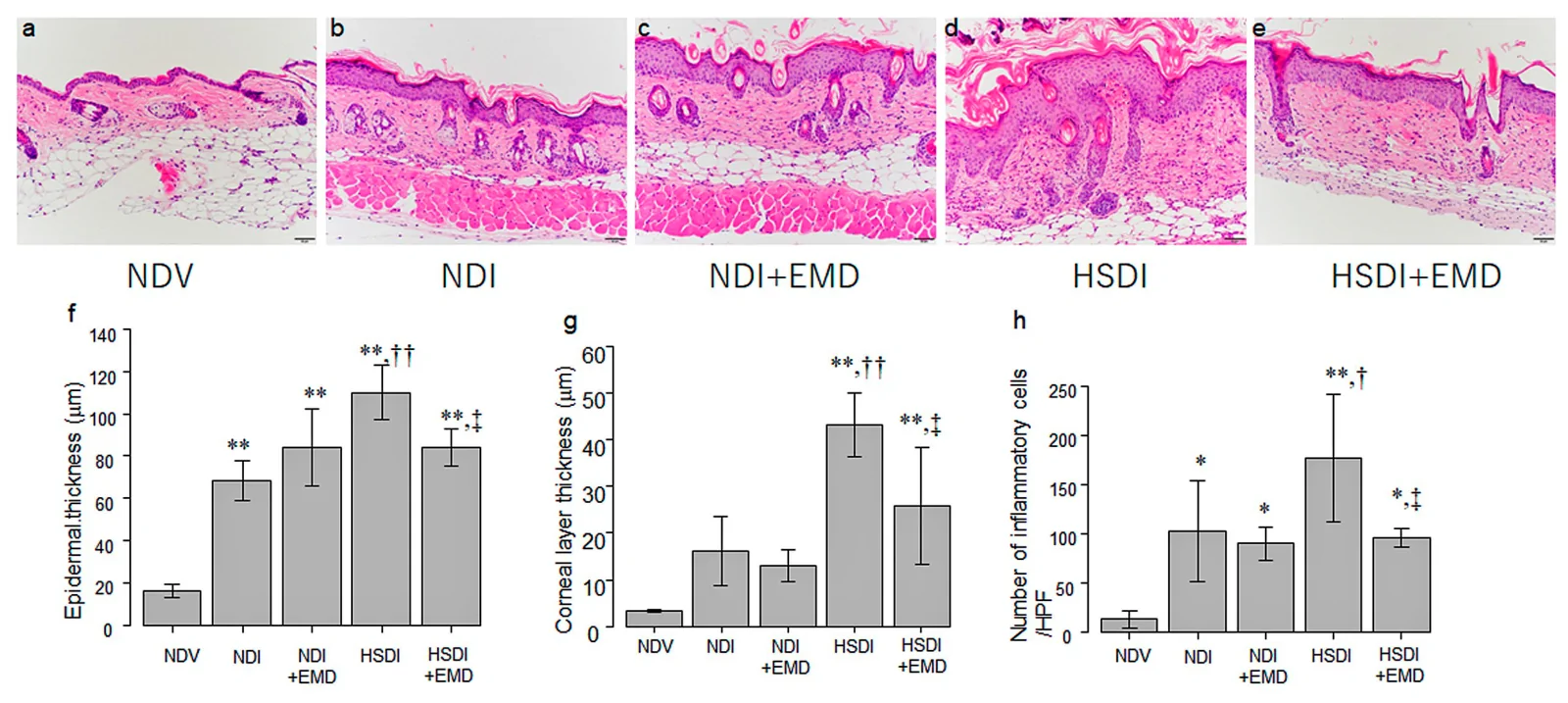
EMD638683 (EMD) reduces the thickness of the epidermis and corneal layers and the number of infiltrating inflammatory cells in imiquimod (IMQ)-induced dermatitis of mice fed with a high-salt diet (HSD). Hematoxylin–eosin staining of normal diet (ND)-fed, Vaseline-treated mice intraperitoneally injected with 1% dimethyl sulfoxide in phosphate-buffered saline (DMSO-PBS) (NDV) (**a**); ND-fed, IMQ-treated, DMSO-PBS-injected mice (NDI) (**b**); ND-fed, IMQ-treated mice injected with EMD in DMSO-PBS (NDI + EMD) (**c**); HSD-fed, IMQ-treated, DMSO-PBS-injected mice (HSDI) (**d**); and HSD-fed, IMQ-treated mice injected with EMD in DMSO-PBS (HSDI + EMD) (**e**). The bar in each panel represents 50 μm. The thickness of the epidermis (**f**) and corneal layers (**g**) and the number of infiltrating cells (**h**) measured or counted under × 400 high-power field (HPF) in five random grids per section are presented as mean ± standard deviation (*n* = 5/group). * *p* < 0.05, ** *p* < 0.01 versus NDV; † *p* < 0.05, †† *p* < 0.01 versus NDI; ‡ *p*< 0.05 versus HSDI, by one-way ANOVA, followed by Tukey post hoc test.

Histologically, EMD638683 decreased the thickness of the epidermis and corneal layers and the number of infiltrating cells in the skin of HSDI mostly to the levels of NDI (Figure 7d–h), while EMD638683 did not significantly reduce those of NDI (Figure 7b,c,f–h).

**Figure 8:**
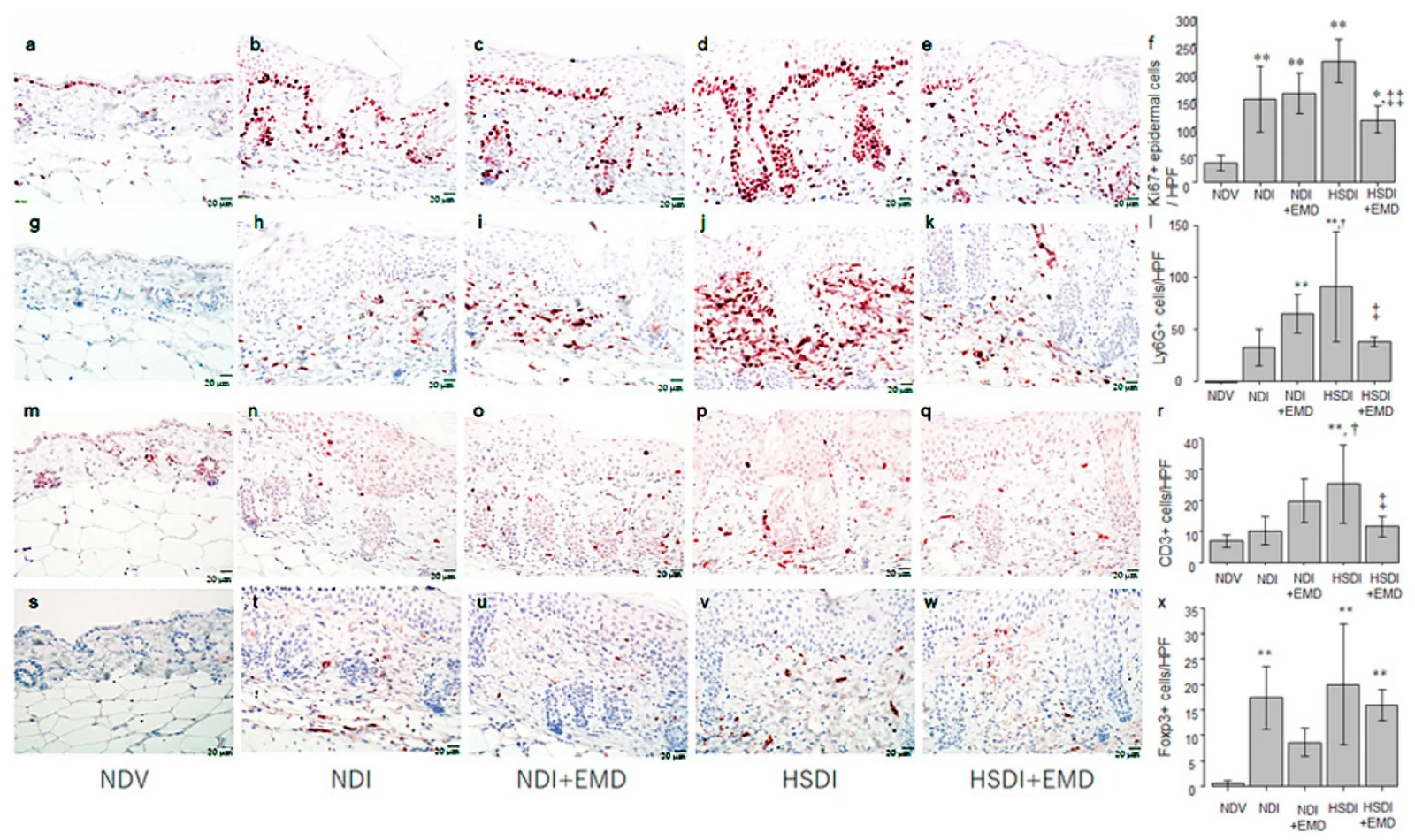
EMD638683 (EMD) reduces the numbers of epidermal Ki67+ cells, infiltrating Ly6G+ cells, and CD3+ cells in imiquimod (IMQ)-induced dermatitis of mice fed a high-salt diet (HSD). Skin sections from normal diet (ND)-fed, Vaseline-treated mice intraperitoneally injected with 1% dimethyl sulfoxide in phosphate-buffered saline (DMSO-PBS) (NDV) (**a**,**g**,**m**,**s**); ND-fed, IMQ-treated, DMSO-PBS-injected mice (NDI) (**b**,**h**,**n**,**t**); ND-fed, IMQ-treated mice injected with EMD in DMSO-PBS (NDI + EMD) (**c**,**i**,**o**,**u**); HSD-fed, IMQ-treated, DMSO-PBS-injected mice (HSDI) (**d**,**j**,**p**,**v**); and HSD-fed, IMQ-treated mice injected with EMD in DMSO-PBS (HSDI + EMD) (**e**,**k**,**q**,**w**) are immunohistochemically stained for Ki67 (**a**–**e**), Ly6G (**g**–**k**), CD3 (**m**–**q**), and Foxp3 (**s**–**w**). The bar in each panel represents 20 μm. The numbers of cells positive for Ki67 (**f**), Ly6G (**l**), CD3 (**r**), or Foxp3 (**x**) counted under × 400 high-power field (HPF) in five random grids per section are presented as mean ± standard deviation (*n* = 5/group). * *p* < 0.05, ** *p* < 0.01 versus NDV; † *p* < 0.05 versus NDI; ‡ *p*< 0.05, ‡‡ *p* < 0.01 versus HSDI, by one-way ANOVA, followed by Tukey post hoc test.

In immunohistochemical analyses, EMD638683 reduced the numbers of Ki67+ proliferating epidermal keratinocytes (Figure 8a–f), Ly6G+ neutrophils (Figure 8g–l), and CD3+ T cells (Figure 8m–r) in the skin of HSDI mostly to the levels of NDI, while EMD638683 did not significantly reduce those of NDI. EMD638683 did not appear to alter the number of Foxp3+ cells in NDI and HSDI (Figure 8s–x). The results strongly suggest that EMD638683 may decrease the number of proliferating keratinocytes, infiltrating neutrophils and T cells in the IMQ-treated skin of HSD-fed mice but not in the IMQ-treated skin of ND-fed mice, indicating that SGK1 may mediate the HSD-induced increase in the above cells.

**Figure 9:**
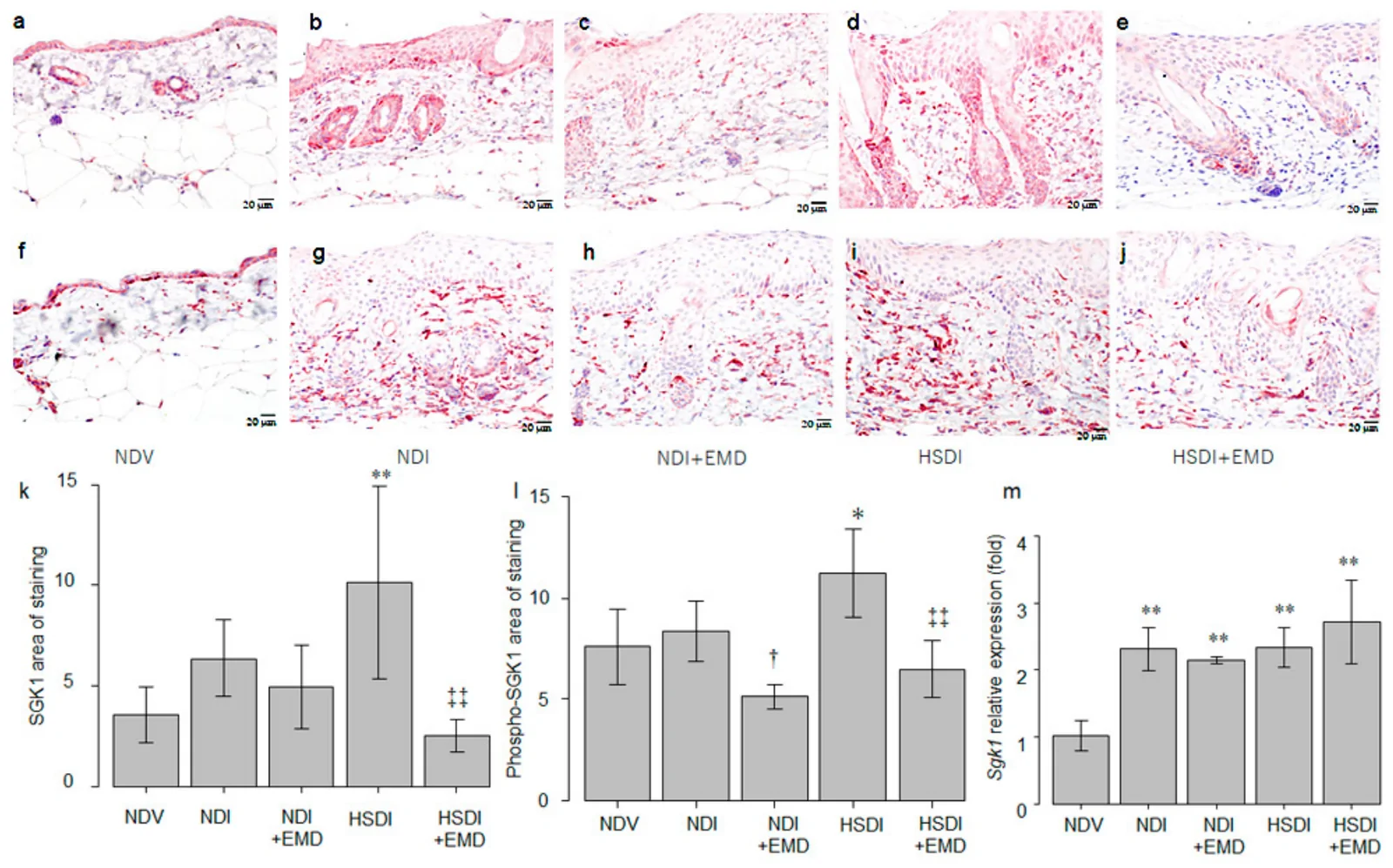
EMD638683 (EMD) reduces the immunostaining areas of whole and Ser 422-phosphorylated forms of SGK1 in imiquimod (IMQ)-induced dermatitis of mice fed a high-salt diet (HSD). Skin sections from normal diet (ND)-fed, Vaseline-treated mice intraperitoneally injected with 1% dimethyl sulfoxide in phosphate-buffered saline (DMSO-PBS) (NDV) (**a**,**f**); ND-fed, IMQ-treated, DMSO-PBS-injected mice (NDI) (**b**,**g**); ND-fed, IMQ-treated mice injected with EMD in DMSO-PBS (NDI + EMD) (**c**,**h**); high-salt diet (HSD)-fed, IMQ-treated, DMSO-PBS-injected mice (HSDI) (**d**,**i**); and HSD-fed, IMQ-treated mice injected with EMD in DMSO-PBS (HSDI + EMD) (**e**,**j**) are immunohistochemically stained for SGK1 (**a**–**e**) or phospho-SGK1 (**f**–**j**). The bar in each panel represents 20 μm. The area of immunostaining for SGK1 (**k**) or phospho-SGK1 (**l**) is presented as mean ± standard deviation (*n* = 5/group). * *p* < 0.05, ** *p* < 0.01 versus NDV; † *p* < 0.05 versus NDI; ‡‡ *p* < 0.01 versus HSDI, by one-way ANOVA, followed by Tukey post hoc test. *Sgk1* mRNA level in the skin was evaluated by qPCR. mRNA levels of *Sgk1* normalized to those of *Gapdh* are shown as fold induction relative to that of NDV, presented as mean ± standard deviation (**m**) (*n* = 6/group).

EMD638683 reduced immunostaining areas of both whole (Figure 9a–e,k) and Ser 422-phosphorylated forms of SGK1 (Figure 9f–j,l) in keratinocytes and dermal-infiltrating cells in IMQ-treated skin of mice fed with HSD while in IMQ-treated skin of ND-fed mice, EMD638683 reduced immunostaining area of phospho-SGK1 (Figure 9l) but not that of whole SGK1 (Figure 9k). EMD638683 did not alter *Sgk1* mRNA levels in IMQ-treated skin of ND- or HSD-fed mice (Figure 9m). These results indicate that activity of SGK1 may contribute to the increase in immunostaining areas of whole and activated forms of SGK1 in IMQ-treated skin of HSD-fed mice.

#### 2.3.2. The Inhibitory Effects of EMD638683 on HSD-Induced Increase in mRNA Levels of Proinflammatory Cytokines/Chemokines and Proliferation-Related Molecules in IMQ-Induced Dermatitis

**Figure 10:**
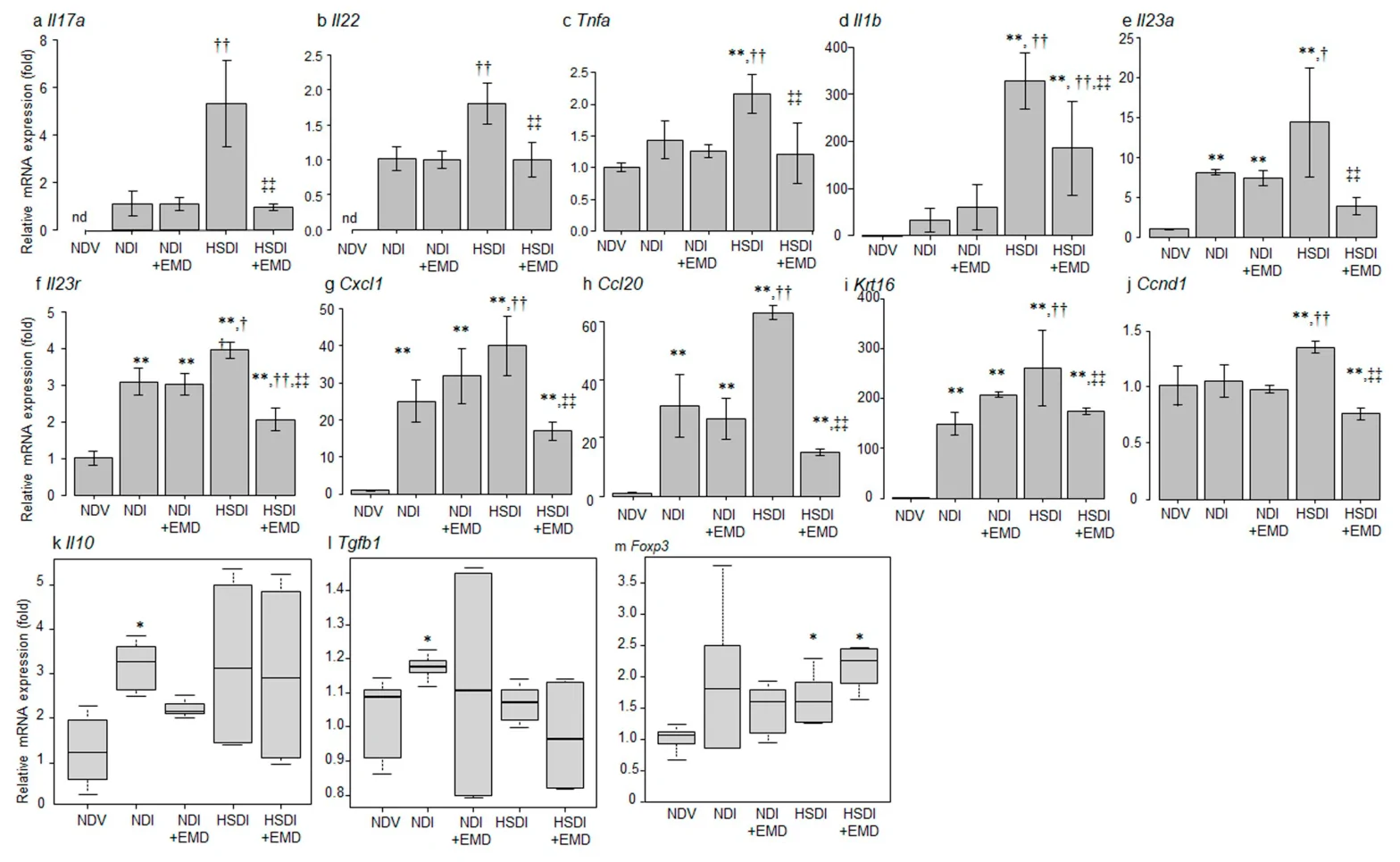
mRNA levels of *Il17a* (**a**), *Il22* (**b**), *Tnfa* (**c**), *Il1b* (**d**), *Il23a* (**e**), *Il23r* (**f**), *Cxcl1* (**g**), *Ccl20* (**h**), *Krt16* (**i**), *Ccnd1* (**j**), *Il10* (**k**), *Tgfb1* (**l**), and *Foxp3* (**m**) in the skin from normal diet (ND)-fed, Vaseline-treated mice intraperitoneally injected with 1% dimethyl sulfoxide in phosphate-buffered saline (DMSO-PBS) (NDV); ND-fed, imiquimod (IMQ)-treated, DMSO-PBS-injected mice (NDI); ND-fed, IMQ-treated mice injected with EMD638683 (EMD) in DMSO-PBS (NDI + EMD); high-salt diet (HSD)-fed, IMQ-treated, DMSO-PBS-injected mice (HSDI); and HSD-fed, IMQ-treated mice injected with EMD in DMSO-PBS (HSDI + EMD), evaluated by qPCR. mRNA levels of individual molecules normalized to those of *Gapdh* are shown as fold induction relative to that of NDV, presented as mean ± standard deviation or median [interquartile range] (*n* = 6/group). *Il17a* and *Il22* mRNA normalized to that of *Gapdh* were not detected in NDV, and their levels are presented as fold induction relative to NDI. * *p* < 0.05, ** *p* < 0.01 versus NDV; † *p* < 0.05, †† *p* < 0.01 versus NDI; ‡‡ *p* < 0.01 versus HSDI, by one-way ANOVA, followed by Tukey post hoc test (**a**–**j**) or by Kruskal–Wallis test followed by Steel Dwass post hoc test (**k**–**m**). nd, not detected.

EMD638683 reduced mRNA levels of *Il17a*, *Il22*, *Tnfa*, *Il1b*, *Il23a*, *Il23r*, *Cxcl1*, and *Ccl20* in HSDI mostly to the levels of NDI; however, it did not significantly alter those of NDI (Figure 10a–h). EMD638683 also reduced mRNA levels of *Krt16* and *Ccnd1* in HSDI mostly to the levels of NDI; however, it did not significantly alter those of NDI (Figure 10i,j). EMD638683 did not appear to alter the mRNA levels of *Il10*, *Tgfb1*, and *Foxp3* in NDI and HSDI (Figure 10k–m). These results suggest that EMD638683 may suppress the HSD-induced increase in mRNA levels for Th17-related proinflammatory or proliferation-related molecules in IMQ-treated skin but may not suppress the above increase by IMQ itself. The results indicate that SGK1 may mediate the HSD-induced increase in the above mRNA levels, at least partially.

## 3. Discussion

In this study, HSD exacerbated murine IMQ-induced psoriasis-like dermatitis, resulting in increased scaling and skin thickening and increased acanthosis, hyperkeratosis, and inflammatory cell infiltration, including neutrophils and T cells. HSD elevated the mRNA levels of inflammatory cytokines/chemokines or their receptor, *Il17a*, *Il22*, *Tnfa*, *Il1b*, *Il23a*, *Il23r*, *Cxcl1*, *Ccl20*, and proliferation-related molecules, *Krt16* and *Ccne1*. These effects of HSD were associated with increased immunostaining areas of whole and activated forms of SGK1 in epidermal keratinocytes and dermal-infiltrating cells. The SGK1 inhibitor EMD638683 mostly reversed the HSD-induced exacerbation of IMQ-induced dermatitis, indicating that SGK1 may at least partially mediate the stimulatory effects of HSD. Since EMD638683 suppressed the HSD-induced increases in *Il17a*, *Il22*, *Il23a*, and *Il23r* mRNA levels, SGK1 may be upstream of the IL-23/IL-17 pathway, possibly increasing the mRNA levels of these molecules in target cells.

HSD increased the immunostaining areas of whole and Ser 422-phosphorylated forms of SGK1 in keratinocytes and dermal-infiltrating cells in IMQ-induced dermatitis. Further quantitative analysis, such as immunoblotting, should clarify whether HSD increases those protein levels. It is reported that high extracellular salt induces the phosphoinositide-3 kinase (PI3K) signaling pathway in target cells [23]. PI3K further activates downstream kinases, mammalian target of rapamycin complex 2 and phosphoinositide-dependent protein kinase 1, which respectively phosphorylate the carboxy-terminal hydrophobic motif and kinase domain of SGK1, leading to the activation of the enzymatic function of SGK1 [24]. The above PI3K/SGK1 cascade might work in IMQ-treated skin of HSD-fed mice. However, the *Sgk1* mRNA levels increased by IMQ were not further enhanced by HSD. The increase in *Sgk1* mRNA may be caused by IMQ alone and may not be influenced by high-salt stimulus. How HSD regulates the activity and expression of SGK1 should be further examined in individual target cells of IMQ-induced dermatitis.

In IMQ-induced dermatitis, IL-17A and IL-22 were mostly derived from Th17 cells or γδT17 cells, while CXCL1 and CCL20 were mainly produced by epidermal keratinocytes [4]. TNF-α and IL-1β were produced by various types of cells, such as DCs, macrophages, keratinocytes, or fibroblasts [4]. The target cell types for the HSD-induced increase in individual cytokines/chemokines should be further identified.

It is reported that high levels of extracellular sodium are transported via ENaCs into APCs, and that the sodium entry activates cytosolic SGK1, which further induces the expression and assembly of ENaCγ and α and allows further entry of sodium via ENaCs [11]. The increased intracellular sodium is exchanged for calcium via sodium–calcium exchangers, leading to the activation of calcium-sensitive protein kinase C, which then activates nicotinamide adenine dinucleotide phosphate oxidase [25], inducing ROS in APCs. The ROS induces the formation of immunogenic isoLG and activates the NLRP3 inflammasome [24]. The isoLG and inflammasome in APCs induce the secretion of IL-1β, IL-23, or TNF-α, and facilitate the presentation of isoLG-protein adducts as neoantigens to T cells [13], which induces T cells to produce proinflammatory cytokines, such as IL-17A or IL-22 [13,25]. The above SGK1/ENaC/ROS/isoLG-inflammasome pathway might be related to the HSD-induced increase in *Il1b*, *Tnfa*, or *Il23* mRNA levels in IMQ-induced dermatitis.

The increased extracellular sodium is also transported into T cells and activates cytosolic SGK1 [26,27]. The activated SGK1 phosphorylates forkhead box protein O1 (FOXO1), which blocks the inhibitory function of FOXO1 to trans-repress the *Il23r* gene, resulting in increased *IL23r* gene expression [16,17,21,27], making T cells responsive to IL-23 together with IL-1β, generating pathogenic Th17 or γδ T17 cells [27]. Since activated SGK1 can activate IκB kinase and promote NF-κB-mediated gene expression [28,29], NF-κB may also be involved in the high-sodium/SGK1-induced *Il17a* or *Il22* mRNA increase in T cells. Taken together, this indicates that SGK1 activated by HSD might inhibit FOXO1 and stimulate NF-κB in T cells, and thus increase *Il23r*, *Il17a*, and *Il22* mRNA levels, since the increase was suppressed by the SGK1 inhibitor EMD638683.

Further, high sodium-induced SGK1 may also affect epithelial cells; it is reported that high extracellular sodium in vitro increased mRNA levels of *Cxcl1* and *Ccl20* in murine keratinocytes [30] or induced the phosphorylation of NF-κB p65 in LPS-stimulated human retina pigment epithelial cells [31]. Similarly to the in vitro studies, in IMQ-induced dermatitis, SGK1 activated by HSD may promote NF-κB-dependent expression of chemokines such as CXCL1 and CCL20, which respectively attract neutrophils and Th17 cells/DCs. It is also reported that SGK1 promotes transcriptional activity of activator protein-1 (AP-1), [32] and that AP-1 transactivates *Krt16* gene expression in cooperation with Sp1 [33]. Thus, in IMQ-induced dermatitis of HSD-fed mice, SGK1-induced AP-1 might contribute to the increase in *Krt16* mRNA levels in epidermal keratinocytes, which may lead to their hyperproliferation and abnormal differentiation, and enhanced skin thickening and scales.

In addition, it has been reported that activated SGK1 phosphorylates the transcription factor cAMP response element-binding protein (CREB) and promotes the CREB-mediated expression of the cell-cycle-progressing gene *Ccnd1*, which encodes cyclin D1, in basilar artery smooth muscle cells [34], leading to the progression of the cell cycle from G0/G1 to S phase and enhancing proliferation. Similar mechanisms might also be involved in the HSD-induced increase in *Ccnd1* mRNA levels and epidermal hyperproliferation observed in this study.

On the other hand, anti-inflammatory roles of SGK1 have also been reported; SGK1 in macrophages may sustain the cell surface expression of NECTIN2, which interacts with TIGIT on the cell surface of Tregs and promotes the immunosuppressive activity of Tregs [35]. SGK1 is known to play either pro- or anti-inflammatory roles in a context-specific manner, depending on the experimental conditions [36,37]; accordingly, the SGK1 inhibitor EMD638683 might either inhibit or stimulate inflammatory diseases. Meng et al. reported results opposite to ours: the SGK1 inhibitor EMD638683 exacerbated IMQ-induced psoriasis-like dermatitis in ND-fed mice and indicated an anti-inflammatory role of SGK1 in suppressing the Bruton’s agammaglobulinemia tyrosine kinase-induced activation of NF-κB [38]. In our study, EMD638683 did attenuate IMQ-induced dermatitis in HSD-fed mice but did not alter it in ND-fed mice. The differences from the study by Meng et al. may be due to the different experimental conditions: they applied 62.5 mg IMQ for 7 days to ND-fed mice, while we applied 25 mg IMQ for 5 days to either ND- or HSD-fed mice. Under high-sodium conditions (such as in an HSD), SGK1 may act as a simulator for psoriasis-like dermatitis induced by suboptimal doses of IMQ. Conversely, under normal sodium conditions (such as in an ND), SGK1 may play an anti-inflammatory role in psoriasis-like dermatitis induced by maximal doses of IMQ, while the anti-inflammatory effects of SGK1 with suboptimal doses of IMQ might be negligible. Since the phospho-SGK1 immunostaining area in NDI was not significantly increased compared to NDV in the present study, the activation of SGK1 and the anti-inflammatory action of SGK1 might be minimal in NDI, and thus the aggravation of dermatitis by EMD638683 in NDI may not be detected. Further studies should examine the pro- or anti-inflammatory roles of SGK1 in IMQ-induced dermatitis under various conditions using different strains, ages, or sexes of mice; different doses or durations of IMQ application; diets containing various concentrations of NaCl; and different doses, routes, and timings of EMD638683 administration.

In relation to our present study, Hu et al. recently reported that an HSD (8% NaCl) aggravated murine IMQ-induced psoriasis-like dermatitis with increased infiltration of CCR6^+^ γδT17 cells [30]. Our present results support their results and additionally clarify that the HSD increased the immunostaining areas of whole and activated forms of SGK1 in the lesional skin and that inhibition of SGK1 attenuated the HSD-induced aggravation of dermatitis. Compared to their study using 60-day feeding with an 8% NaCl diet, 21-day feeding with a 4.2% NaCl diet did exacerbate IMQ-induced dermatitis in our present study. The present results suggest that even short-term, modestly excessive salt intake may exacerbate psoriasis-like dermatitis in mice models.

This study has some limitations. Firstly, we used only IMQ-induced dermatitis as a psoriasis mouse model. The effects of HSD should be further evaluated in different psoriasis models, such as keratinocyte-specific gene-manipulated mice (K14-VEGF, K5.Stat3C), intradermal IL-23-injected or IL-23 mini-circle DNA-delivered mice. Secondly, other mechanisms independent of SGK1 might be involved in the HSD-induced exacerbation of IMQ-induced dermatitis; HSD in mice activated salt-regulated Ste20-related proline/alanine-rich kinase, which induced IL-21 expression in CD4+ T cells and exacerbated autoimmune diabetes [39]. HSD in mice reduced the abundance of anti-inflammatory L-fucose-releasing bacteria, *Candidatus arthromitus*, in the gut microbiome, leading to the exacerbation of zymosan-induced peritonitis [40]; alternatively, HSD reduced the abundance of indole-3 acetic acid-releasing *Lactobacillus murinus* in the gut microbiome, leading to the exacerbation of experimental autoimmune encephalomyelitis [41]. Further studies should elucidate if the above mechanisms may also be involved in HSD-induced exacerbation of IMQ-induced dermatitis. Thirdly, we performed immunohistochemical staining of total and phospho-SGK1 to see which cell types show the activation of SGK1. However, future studies should clarify whether HSD increases those protein levels, using quantitative methods such as immunoblotting. Fourthly, we have not clarified the transcription factors involved in the HSD-induced increase in mRNAs for pro-inflammatory or growth-stimulatory molecules, such as NF-κB, STAT3, AP-1, or CREB, and these should be analyzed in further studies. Fifthly, we did not examine the effects of HSD on mRNA levels of proinflammatory molecules in individual target cells such as keratinocytes, DCs, or T cells in the lesional skin. The overexpressed molecules in each target cell should be identified by future studies using single-cell RNA sequencing in IMQ-applied skin and also by investigating in vitro effects of high salt and involvement of SGK1 in individual cell lines. Lastly, the present study examined whether the SGK1 inhibitor EMD638683 blocks the HSD-induced exacerbation of IMQ-induced dermatitis to see the involvement of SGK1 in the effects of HSD. However, further studies should examine whether *sgk1* gene knockout cancels the stimulatory effects of HSD on IMQ-induced dermatitis to establish a causal relationship between SGK1 and the effects of HSD.

## 4. Materials and Methods

### 4.1. Mice

BALB/c mice were obtained from CLEA Japan, Inc. (Tokyo, Japan). All mice for experiments were 6-week-old females and were maintained in specific pathogen-free conditions at the animal facility of Nippon Medical School.

### 4.2. Reagents

Rabbit monoclonal anti-mouse CD3 (SP7) was obtained from Abcam (Cambridge, UK). Rat monoclonal anti-mouse Ly6G (1A8) was obtained from Aviva Systems Biology, Corp. (San Diego, CA, USA). Rat monoclonal anti-mouse Foxp3 and rabbit polyclonal anti-Ki-67 were obtained from Thermo Fisher Scientific (Waltham, MA, USA). Rabbit monoclonal anti-SGK1 antibody and rabbit polyclonal anti-phospho-SGK1 (Ser 422) were obtained from Abcam (Waltham, MA, USA). EMD638683 was obtained from Selleck Chemicals (Houston, TX, USA).

### 4.3. Development of IMQ-Induced Psoriasis-like Dermatitis

Mice were fed either an HSD (D24051401, 4.2% NaCl; Research Diets, New Brunswick, NJ, USA) plus 1% NaCl-containing drinking water or an ND (D14071802, 0.2% NaCl; Research Diets) plus NaCl-free drinking water for 3 weeks. Then, 25 mg of 5% IMQ cream (Beselna cream; Mochida Pharmaceuticals, Tokyo, Japan) or Vaseline (control) was applied to the shaved back skin for 5 consecutive days (days 0–4). A blinded investigator performed daily evaluations for clinical severity scoring based on the psoriasis area and severity index. Erythema, scale, and thickness were each scored independently on a scale from 0 to 4 (0, none; 1, slight; 2, moderate; 3, marked; 4, very marked), and the sum of the scores was used as the total clinical score (0–12) [8]. Each group consisted of three mice, and a series of experiments was done 4 times. In another series of experiments done 3 times, mice were fed an ND or HSD as above for three weeks, then 0.2 mg EMD638683 in 1% dimethyl sulfoxide (DMSO)-containing phosphate-buffered saline (PBS) (DMSO-PBS) or 1% DMSO-PBS 500 μL, as a control, was intraperitoneally injected daily one hour before IMQ or Vaseline application from day −1 to day 3.

### 4.4. Histological Analyses

On day 5, murine back-skin samples were obtained, and half of the sample was paraffin-embedded for hematoxylin–eosin staining, while the other half was embedded in Optimal Cutting Temperature Compound (Sakura Finetek Japan, Tokyo, Japan) and snap-frozen in liquid nitrogen for immunohistochemical staining. The frozen samples were cut into 5 μm thick sections, fixed with cold acetone, and washed with PBS. Samples were incubated with blocking solution (3.75% bovine serum albumin/5% goat serum, Zymed, Carlsbad, CA, USA) for 30 min, and incubated overnight at 4 °C with the primary antibodies (anti-CD3, Ly6G, Ki67, Foxp3, SGK1, or phospho-SGK1 [S422] antibodies) (Table 1) or isotype controls in appropriate dilutions. After washing, slides were incubated with the secondary antibodies for 2 h. The slides were washed again and incubated with an avidin–biotin peroxidase complex followed by Nova RED (Vector Labs, Burlingame, CA, USA), and counterstained with Mayer’s hematoxylin. The thickness of the epidermis and corneal layer was measured, and numbers of inflammatory infiltrating cells and Ki67+, CD3+, Ly6G+, or Foxp3+ cells were counted under x 400 high-power fields in five random grids per section. The Image J Fiji software version 1.54. was used for semi-quantitative analyses of immunohistochemical staining of SGK1 or phospho-SGK1 [42], and the area of staining was measured [43].

### 4.5. Quantitative Real-Time Polymerase Chain Reaction (qPCR)

Total RNA was isolated from murine back-skin samples at day 5 using Nucleospin RNA (Takara Bio Inc., Tokyo, Japan), and cDNA was synthesized using High-Capacity RNA-to-cDNA™ Kit (Thermo Fisher Scientific, Waltham, MA, USA). Gene expression of each sample was quantified in duplicate or triplicate using the TaqMan™ Fast Advanced Master Mix (Thermo Fisher Scientific, Waltham, MA, USA). Primers were purchased from Thermo Fisher Scientific (Table 1). Gene expression levels were normalized to those of *Gapdh* gene mRNA levels as an internal control. *Gapdh* mRNA levels did not change with the treatments with IMQ or HSD (Figure S1). The relative expression levels of each gene were determined by the 2^−ΔΔCT^ method.

### 4.6. Statistical Analyses

Data are presented as mean ± standard deviation for variables with normal distribution, whereas median [interquartile range] for variables with non-parametric distribution. Comparisons were made using one-way analysis of variance (ANOVA) followed by the Tukey post hoc test for variables with normal distribution, whereas the Kruskal–Wallis test was used, followed by the Steel Dwass post hoc test, for variables with non-parametric distribution. Values of *p* < 0.05 represent significant differences.

## 5. Conclusions

HSD exacerbated IMQ-induced psoriasis-like dermatitis, demonstrating increased scaling and skin thickening and enhancement of epidermal thickness, hyperkeratosis, and dermal inflammatory infiltrates, including Ly6G+ neutrophils and CD3+ T cells. HSD increased mRNA levels of Th17-related proinflammatory molecules, *Il17a*, *Il22*, *Tnfa*, *Il1b*, *Il23a*, *Il-23r*, *Cxcl1*, *Ccl20*, and proliferation-related molecules, *Krt16* and *Ccnd1*, in IMQ-induced dermatitis. HSD increased the immunostaining areas of whole and activated forms of SGK1 in epidermal keratinocytes and dermal infiltrating cells. Intraperitoneally administered SGK1 inhibitor EMD638683 blocked the above effects of HSD, indicating the involvement of SGK1 in the stimulatory effects of HSD. The present findings in a mouse model of psoriasis indicate the possibility that HSD contributes to the development or exacerbation of psoriasis in humans, and that SGK1 activation might be involved in those effects. Further studies should seek evidence for HSD-induced exacerbation of symptoms—and their improvement by salt restriction—in patients with psoriasis.

## Figures and Tables

**Table 1:** List of agents.

List of Antibodies Used in Immunohistochemistry			
**Antibody**	**Clone**	**Company**	**Order Number**
Anti-CD3	SP7	Abcam (Cambridge, UK)	67849
Anti-Ly6G	1A8	Aviva Systems (San Diego, CA, USA)	OATA00269
Anti-Foxp3	FJK-16s	Thermo Fisher Scientific(Waltham, MA, USA)	14-5773-82
Anti-Ki67	Polyclonal	Thermo Fisher Scientific(Waltham, MA, USA)	PA5-19462
Anti-SGK1	Y238	Abcam (Waltham, MA, USA)	ab32374
Ati-phospho-SGK1 (S422)	Polyclonal	Abcam (Waltham, MA, USA)	ab55281
TaqMan^®^Gene Expression Assay IDs of genes used in quantitative real-time polymerase chain reaction			
**Gene**	**Assay ID**	**Gene**	**Assay ID**
*Il22*	Mm00444241_m1	*Il17a*	Mm00439618_m1
*Il1b*	Mm00434228_m1	*Krt16*	Mm01306670_g1
*Gapdh*	Mm99999915_g1	*Ccl20*	Mm01268754_m1
*Il10*	Mm01288386_m1	*Cxcl1*	Mm04207460_m1
*Tgfb1*	Mm01178820_m1	*Foxp3*	Mm00475162_m1
*Tnfa*	Mm00443258_m1	*Ccnd1*	Mm00432359_m1
*Sgk1*	Mm00441380_m1	*Il23r*	Mm00519943_m1
*Il23a*	Mm00518984_m1		

## Data Availability

The raw data supporting the conclusions of this article will be made available by the authors upon request.

## Supplementary Materials

The following supporting information can be downloaded at: https://www.mdpi.com/article/10.3390/ijms27188151/s1.

## Abbreviations

The following abbreviations are used in this manuscript:

ANOVA	analysis of variance
AP-1	activator protein-1
APC	antigen-presenting cell
CREB	cAMP response element-binding protein
DC	dendritic cell
DMSO	dimethyl sulfoxide
ENaC	epithelial sodium channel
FOXO1	forkhead box protein O1
HSD	high-salt diet
HSDI	high-salt diet-fed and imiquimod-treated mice
IL	interleukin
IMQ	imiquimod
isoLG	isolevuglandin
LPS	lipopolysaccharide
NDI	normal diet-fed and imiquimod-treated mice
NDV	normal diet-fed and Vaseline-treated mice
NLRP3	nucleotide-binding oligomerization domain-like receptor family pyrin domain containing 3
PBS	phosphate-buffered saline
qPCR	quantitative real-time polymerase chain reaction
PI3K	phosphoinositide-3 kinase
ROS	reactive oxygen species
Th17	type 17 helper T
SGK1	serum- and glucocorticoid-inducible kinase 1
TNF	tumor necrosis factor
Treg	regulatory T cell
