# Supplementary material for: High-Salt Diet Exacerbates Murine Imiquimod-Induced Psoriasis-like Dermatitis via Activation of Serum- and Glucocorticoid-Inducible Kinase 1
Source: Int J Mol Sci. 2026 Sep 13;27(18):8151. doi: 10.3390/ijms27188151 (PMC13607775; doi:10.3390/ijms27188151)
Supplement: Supplementary file 1 [file ijms-27-08151-s001.zip › ijms-4538323-supplementary/Figure S1.pdf]

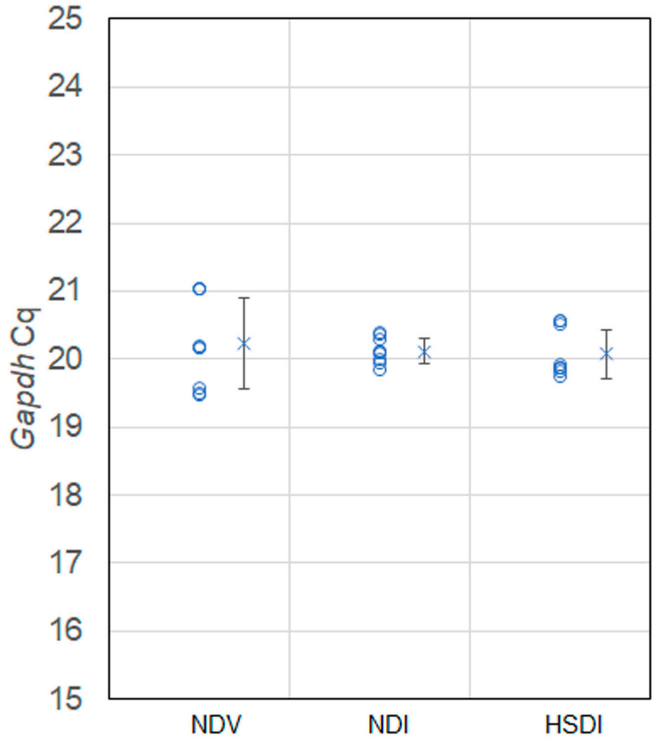

Figure S1. Quantification cycle (Cq) values for *Gapdh* mRNAs evaluated by qPCR.
Total RNA was isolated from murine back skin samples from normal diet (ND)-fed, Vaseline-treated mice (NDV), ND-fed, imiquimod (IMQ)-treated mice (NDI), and high salt diet-fed, IMQ-treated mice (HSDI), at day 5, and converted to cDNA,
cDNA and *Gapdh* primers were used to perform qPCR, and *Gapdh* Cq values were compared among treatment groups. Each group contains three mice and qPCR was performed in triplicate. *Gapdh* Cq of each group is shown as individual values with mean ± standard deviation (n = 9/group). There were no significant differences in *Gapdh* Cq between groups, as evaluated by one-way analysis of variance (p = 0.735).
